# Inhibiting Th1/2 cells influences hepatic capillarization by adjusting sinusoidal endothelial fenestrae through Rho-ROCK-myosin pathway

**DOI:** 10.18632/aging.202425

**Published:** 2021-02-01

**Authors:** Yuesi Zhong, Mingxing Xu, Jingxiong Hu, Xi Huang, Nan Lin, Meihai Deng

**Affiliations:** 1 Department of Hepatobiliary Surgery, The Third Affiliated Hospital of Sun Yat-sen University, Guangzhou 510630, Guangdong, China

**Keywords:** T helper cell, liver sinusoidal endothelial cell, p-MLC, immunity, hepatic capillarization

## Abstract

CD4^+^ T cells are considered to be vital in chronic liver diseases, but their exact roles in hepatic capillarization, the typical characteristic of liver fibrosis, are poorly understood. This study aimed to assess the roles of typical subtype of CD4^+^ T cells, named T helper 1 (Th1) and Th2 cells in liver fibrosis. Taking advantage of well established fibrotic rat model, we conducted *in vitro* and *in vivo* experiments to explore the interactions between liver sinusoidal endothelial cells (LSECs) and Th1/2 cells; meanwhile we evaluated the degree of hepatic capillarization when inhibiting these interactions with inhibitory antibodies. Our results showed that prohibiting interactions between Th2 cells and LSECs caused the restoration of fenestrae, increased cytokine level of Th1 cells and reduction of hepatic capillarization; inhibiting the interaction between Th1 cells and LSECs produced the opposite effects. Moreover, increased Rho and myosin light chain phosphorylation were observed when Th1 cells were inhibited with the corresponding inhibitory antibody; Th2 cell inhibition yielded the opposite results. This study indicated that Th1/2 cells steer the capillarization process in different directions and this effect is probably mediated by the Rho-Rho kinase (ROCK)-myosin signaling pathway.

## INTRODUCTION

Liver fibrosis results from a variety of chronic damages, such as viral hepatitis, metabolic diseases, and nonalcoholic fatty liver disease [1–3], and hepatic capillarization is the characteristic manifestation during this process. Numerous factors influence the occurrence and development of hepatic capillarization, and among them, immune regulation is a core one [4]. Infiltration of T cells, particularly the CD4^+^ T cell subpopulation, was reported to be vital during fibrosis in humans [5]. It was reported that homeostasis of CD4^+^ T cells is pivotal in liver fibrosis [6, 7]. T helper (Th) cells, particularly classical Th1 and Th2 cells, can profoundly influence the fibrotic response and dominate the development of liver fibrosis [8, 9]. However, the ways in which Th1/2 cells affect hepatic capillarization and the mechanism underlying these effects remain largely unknown. In addition, the contribution of adaptive immunity in liver fibrosis is poorly understood.

Liver sinusoidal endothelial cells (LSECs) are microvascular endothelial cells in the liver which possess unique immunological characteristics. Many studies have confirmed that LSECs could be potent antigen-presenting cells priming naïve CD4^+^ T cells and affecting cytokine secretion of Th cells [10, 11]. The mechanism that mediated the recruitment of Th1/2 cells to LSECs was controversial until Bonder C et al*.* verified in 2005 that Th1 and Th2 cells adhered to LSECs via integrin α4 and vascular adhesion protein (VAP)-1, respectively [12], which was totally different from the proven selectin-dependent recruitment paradigm [13, 14].

Sinusoidal endothelial fenestrae (SEF), which commonly arranged in sieve plate-like pores under normal conditions was the unique morphological structure of LSECs. These pores commonly lack diaphragm and basal lamina, therefore, they are viewed as open channels between sinusoidal lumen and the space of Disse, mediating the exchange in hepatic sinusoids [15, 16]. However, when chronic liver damage cannot be eliminated, the LSECs will undergo defenestration, which was characterized by the formation of basement membrane and decrease in the number of SEF [17, 18]. SEF is a type of dynamic structure, its diameter and number may vary in response to different substances and circumstances [19]. Changes of cytoskeleton and Rho signaling pathway exerted critical influence in modulating SEF in liver fibrosis [20–22].

In this study, we reported that Th1/2 cells can actively interact with LSECs in fibrotic rats. We found that inhibiting the interactions can alter the process of hepatic capillarization, and this effect probably relied on cytoskeletal change of LSECs through the Rho-ROCK-myosin signaling pathway.

## RESULTS

### Establishment of fibrotic rat model

**Figure 1:**
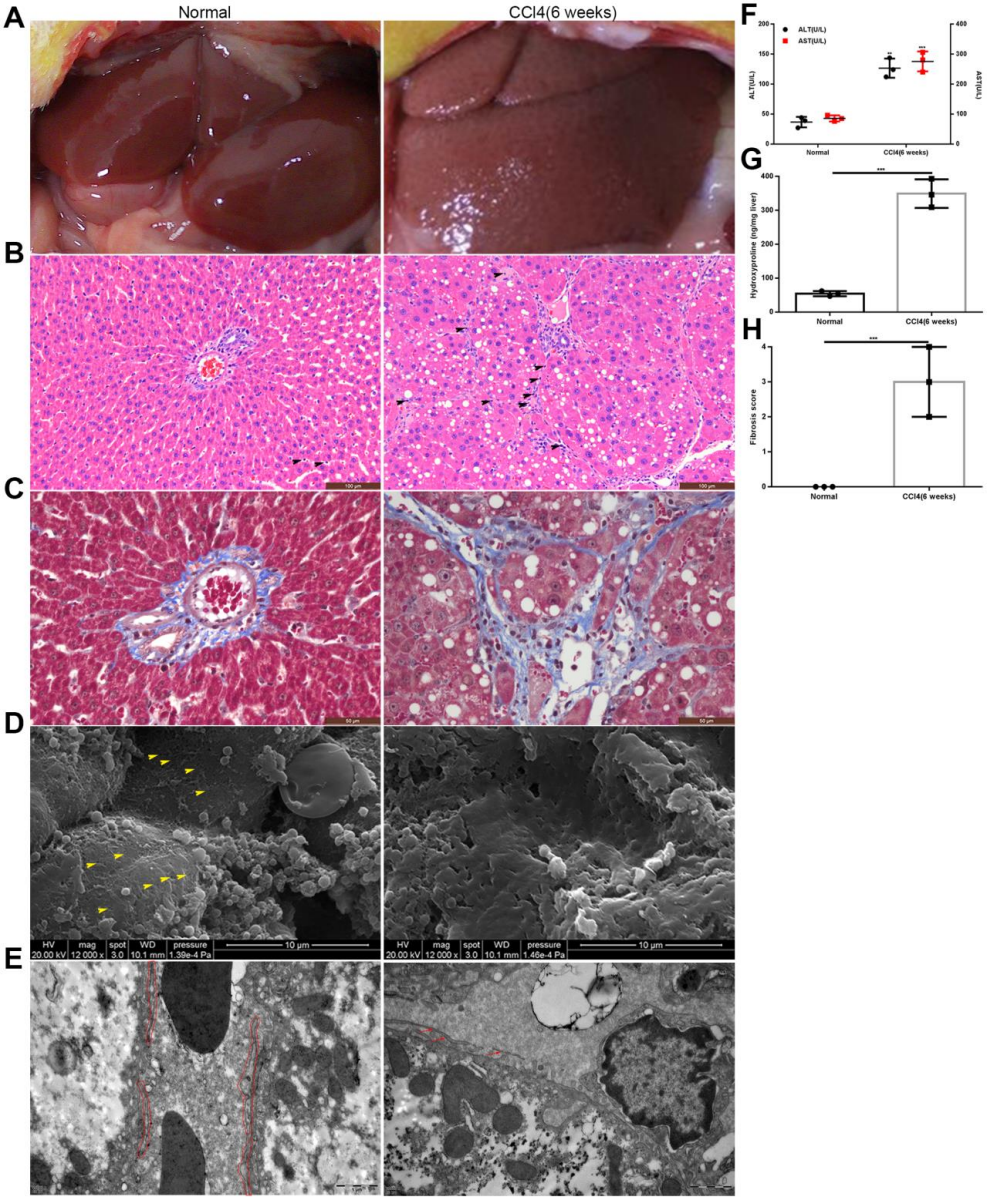
**Establishment of the rat model of liver fibrosis.** (**A**) General appearance of liver in the model group and the normal control group. (**B**, **C**) HE and Masson staining of liver tissues from the model group and normal control group: inflammatory cells accumulated in the hepatic sinusoids and collagen fibers deposited in the space of Disse (the black head of arrow indicates infiltrating lymphocytes). (**D**) SEM: defenestration changes in the model group compared with those in the normal control group (the yellow head of arrow indicates fenestrae). (**E**) TEM: formation of a basement membrane in the model group compared with that in the normal control group (the red irregular area in the normal control group indicated LSECs; and in the model group, the red arrow showed the discontinuous basement membrane). (**F**) Transaminase levels in the model group and the normal control group. (**G**) Hydroxyproline content in the model group and the normal control group. (**H**) Liver fibrosis score in the model group and the normal control group. **p < 0.01, ***p < 0.001.

We established fibrotic rat model by the intraperitoneal injection of 50% CCl_4_ dissolved in oil. Six weeks later, the edge of the liver was blunt, and the hepatic surface was grainy (Figure 1A). Histochemical staining showed numerous inflammatory cells accumulating in the hepatic sinusoids, and collagen fibers were deposited in the space of Disse (Figure 1B, 1C). Scanning electron microscopy (SEM) and transmission electron microscopy (TEM) revealed typical characteristics of liver capillarization, namely defenestration and basement membrane formation (Figure 1D, 1E). The levels of both alanine aminotransferase (ALT) and aspartate transaminase (AST) were significantly higher in fibrotic rats than in normal control (NC) group (Figure 1F). The content of hydroxyproline was approximately 346.5±7.086 ng/mg liver tissue in fibrotic rats, which was much higher than that in NC group (Figure 1G). Moreover, the liver fibrosis score was higher in fibrotic rats than that in normal rats (Figure 1H). Taken together, these results indicated the successful establishment of the liver fibrosis model.

### Th cells regulate liver fibrosis by interacting with LSECs in *in vivo* experiments

**Figure 2:**
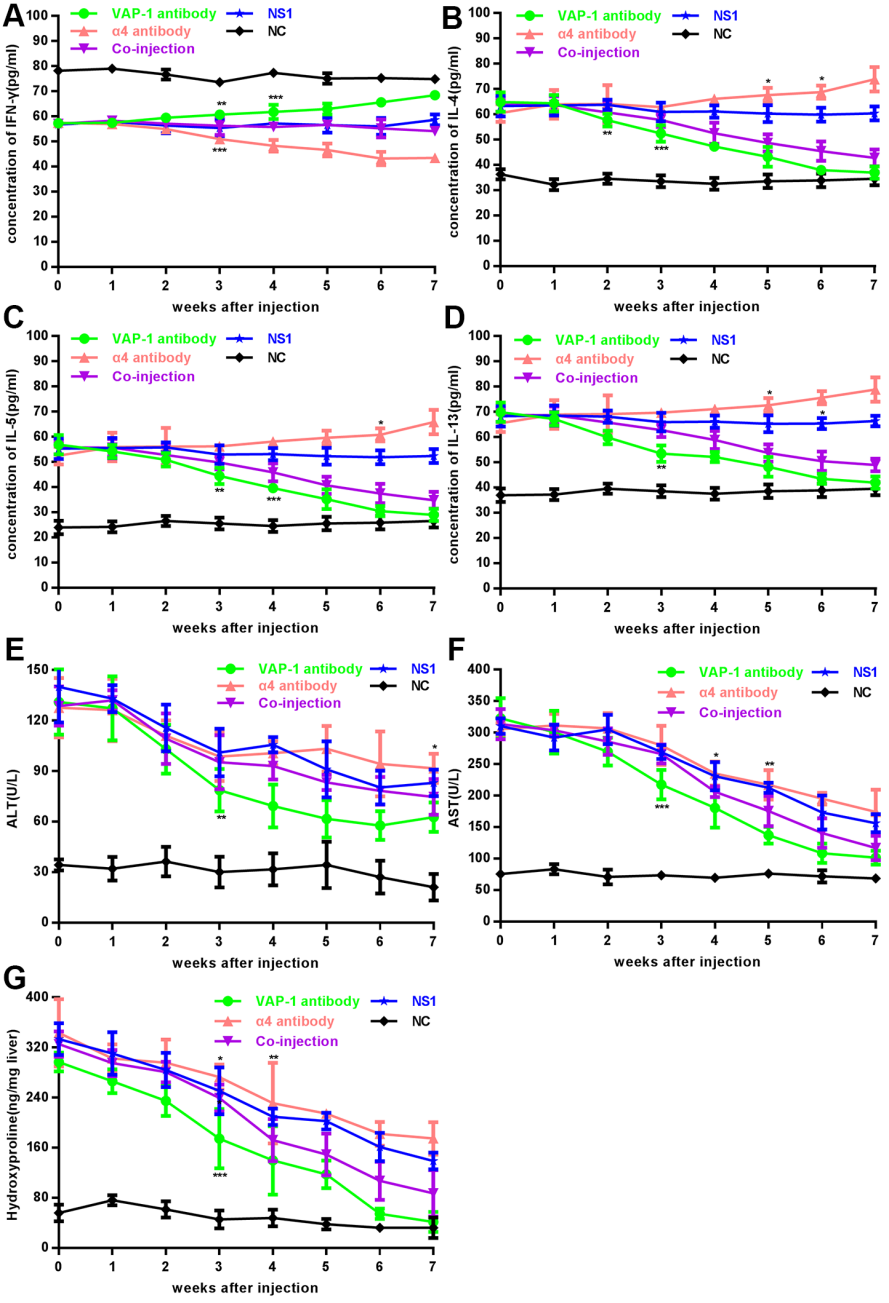
**Changes of cytokines, transaminase and hydroxyproline content in each group in *in vivo* experiments.** (**A**–**F**) Analysis of cytokines (IFN-γ, IL-4, IL-5 and IL-13) and serum ALT, AST in each experimental group and each control group. (**G**) Analysis of hydroxyproline content in each group. At least 3 rats could be used to repeat the experiment at each week point. *p < 0.05, **p < 0.01, ***p < 0.001.

In *in vivo* experiments, we detected levels of cytokines after the injection of inhibitory antibodies. Through observation for 7 consecutive weeks, we found that secretion of IFN-γ, the signature cytokine of Th1 cells, showed an increasing trend in the anti-VAP-1 group and on the 3^rd^ week after injection, it increased to a significant high level; however, a decreasing trend was observed in the anti-integrin α4 group and the level decreased to a significant low value at the 3^rd^ week after injection. In the co-injection group and the two control groups (irrelevant isotype-matched control antibody group (NS1) and NC), no significant changes were detected over the observation period (Figure 2A). Similarly, the expression of IL-4, IL-5 and IL-13, the signature Th2 cytokines showed decreasing trend in the anti-VAP-1 group but an increasing trend in the anti-integrin α4 group (Figure 2B–2D). Compared with initial level, the transaminase (ALT and AST) dropped to a significantly lower level on the 3^rd^ week in the anti-VAP-1 group, however it showed a much less decreasing extent in the anti-integrin α4 group (Figure 2E, 2F). The hydroxyproline content was initially high in each experimental group; however, on the 3^rd^ week after anti-VAP-1 injection, the content began to decrease significantly. And similarly, the decreasing extent was much less in the anti-integrin α4 group (Figure 2G). To further demonstrate the specificities of anti-VAP-1 monoclonal antibody (mAb) and anti-integrin α4 mAb, we compared each experimental group to the NS1 group according the cytokine level of Th cells at the 3^rd^ week after injection, and the results showed a good specificity (Supplementary Figure 1).

**Figure 3:**
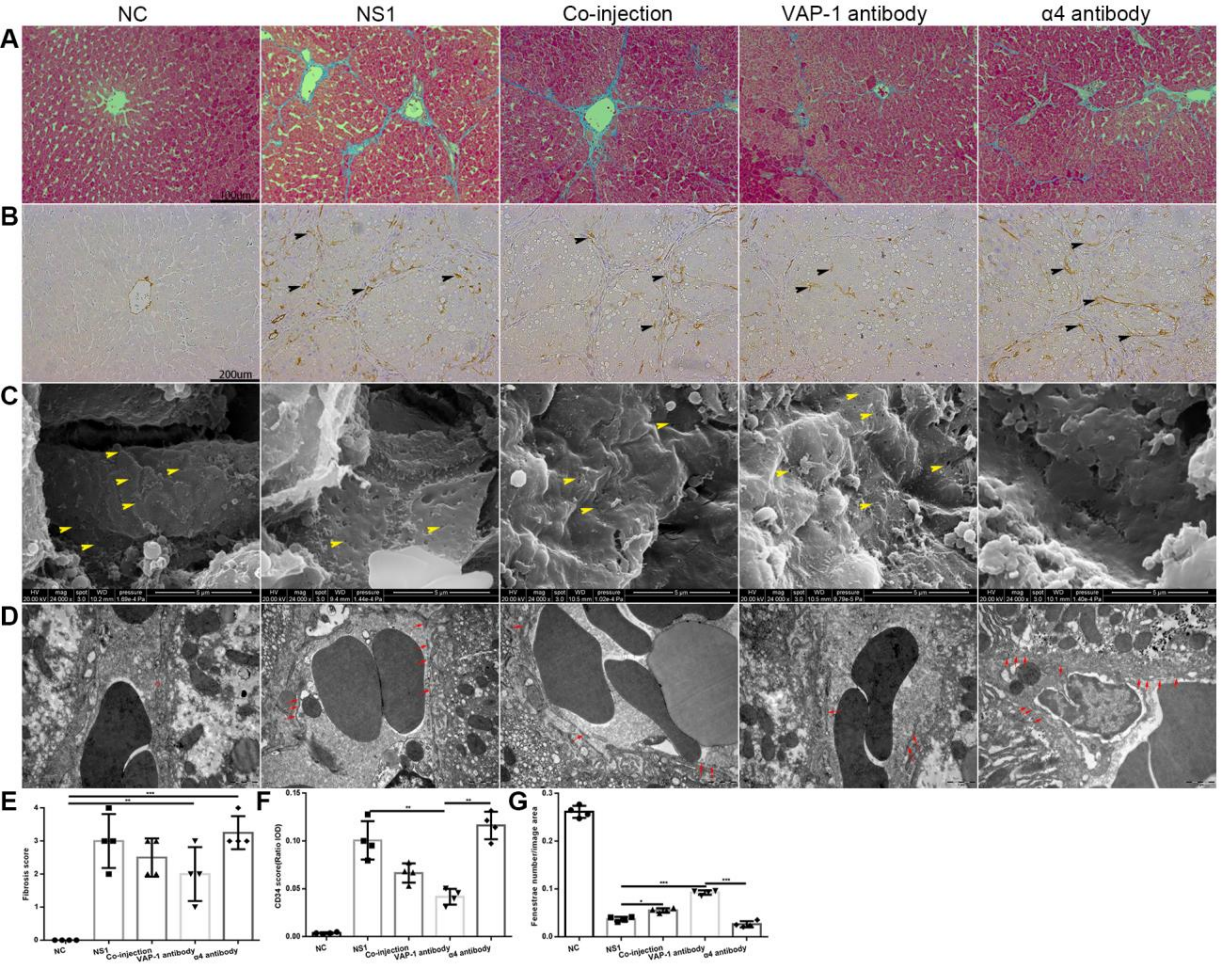
**Morphological change in each group in the *in vivo* experiments.** (**A**) Collagen fiber distribution in each group. (**B**) CD34 expression in each group (the black head of arrow indicates CD34). (**C**) Changes of fenestrae in each group (the yellow head of arrow indicates fenestrae on the LSECs). (**D**) Changes in the basement membrane between the experimental groups and the control groups (the red circle in the NC group indicated the fenestrae on LSECs, and red arrow in other groups showed the basement membrane). (**E**) Liver fibrosis score of each group in (**A**). (**F**) Quantification of CD34 expression of each group in (**B**). (**G**) Quantification of fenestrae number of each group in (**C**). *p < 0.05, **p < 0.01, ***p < 0.001.

From these results, we concluded that external intervention with inhibitory antibodies exerted substantial effects beginning on about the 3^rd^ week after administration. Therefore, we investigated the changes of morphological structure in each group on the 3^rd^ week after injection and found that, as shown in Figure 3A, 3E, the fibrosis score in the anti-VAP-1 group was much lower than that in the other experimental groups; however, in the anti-integrin α4 group, the score was still at a high level. CD34 is an endothelial adhesion molecule, which is not expressed on normal LSECs but is usually viewed as a capillarization marker in liver fibrosis [12, 23, 24]. By immunohistochemical staining, we showed that CD34 expression in the anti-VAP-1 group was significantly lower than that in the other experimental groups, indicating that inhibiting the adhesion of Th2 cells to LSECs with the inhibitory anti-VAP-1 antibody could dramatically prevent the progression of hepatic capillarization or even fibrosis to some extent, but inhibiting the adhesion of Th1 cells to LSECs with the inhibitory anti-integrin α4 antibody exerted the opposite effects (Figure 3B, 3F). Similarly, we investigated defenestration and basement membrane formation in each group and observed that compared with other treatments, injection with the anti-VAP-1 antibody had the greatest effect on reversing the process of defenestration and reducing basement membrane deposition (Figure 3C, 3D, 3G).

Taken together, these results suggested that preventing the adhesion of Th cells to LSECs affects the progression of hepatic capillarization. Moreover, inhibiting Th2 lymphocyte adhesion to LSECs can reduce or even reverse the extent of liver fibrosis to some extent.

### Th cells regulate fenestrae by interacting with LSECs in *in vitro* experiments

**Figure 4:**
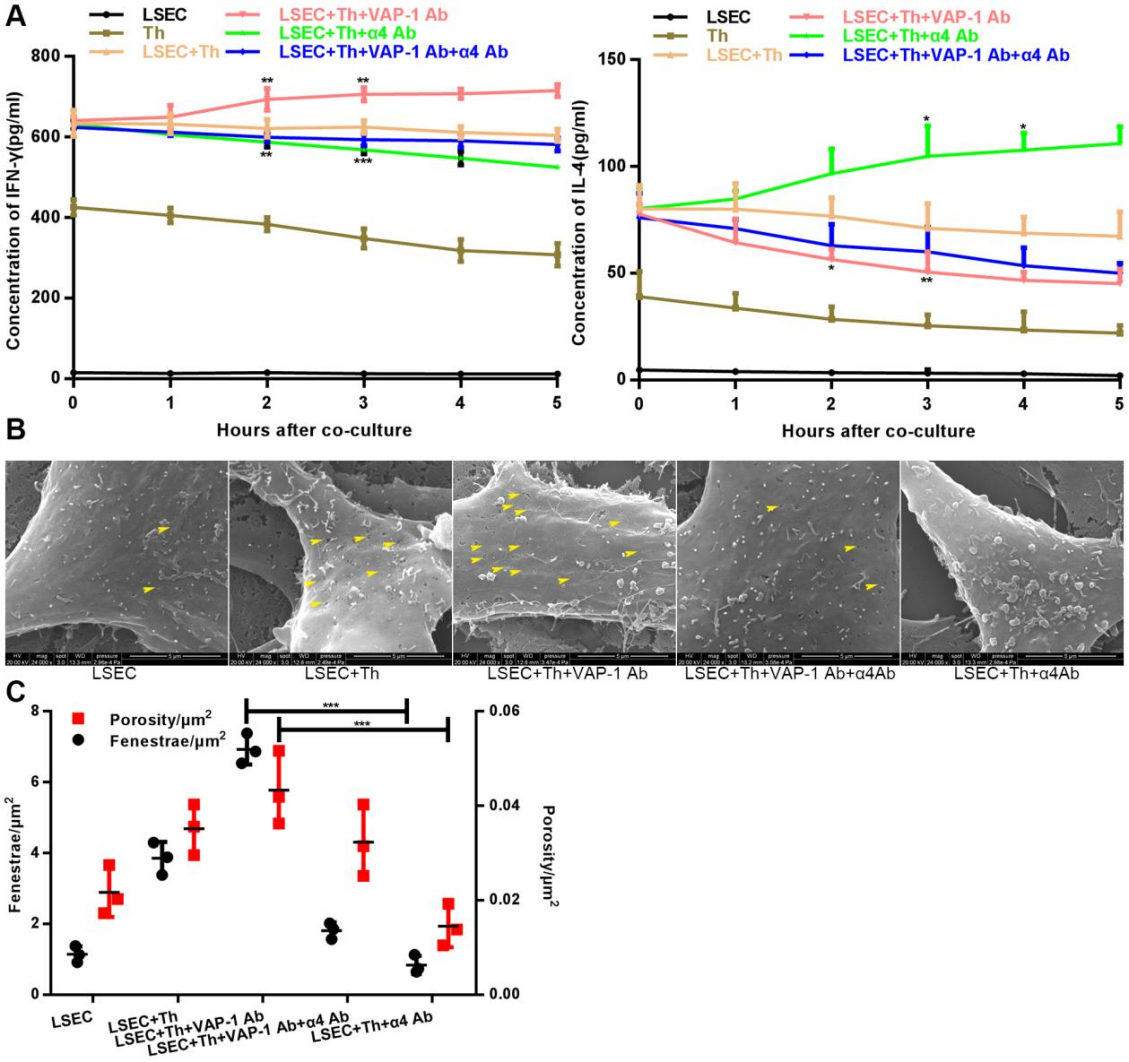
**Changes of cytokine levels and changes of LSEC structure in the *in vitro* coculture experiments.** (**A**) Analysis of cytokines (IFN-γ and IL-4) levels at different coculture time points. (**B**) Distribution and size variance of fenestrae in each group after coculture for 3 h (the yellow head of arrow indicates fenestrae on the LSECs). (**C**) Quantification of fenestrae number and porosity of each group in (**B**). *p < 0.05, **p < 0.01, ***p < 0.001.

**Figure 5:**
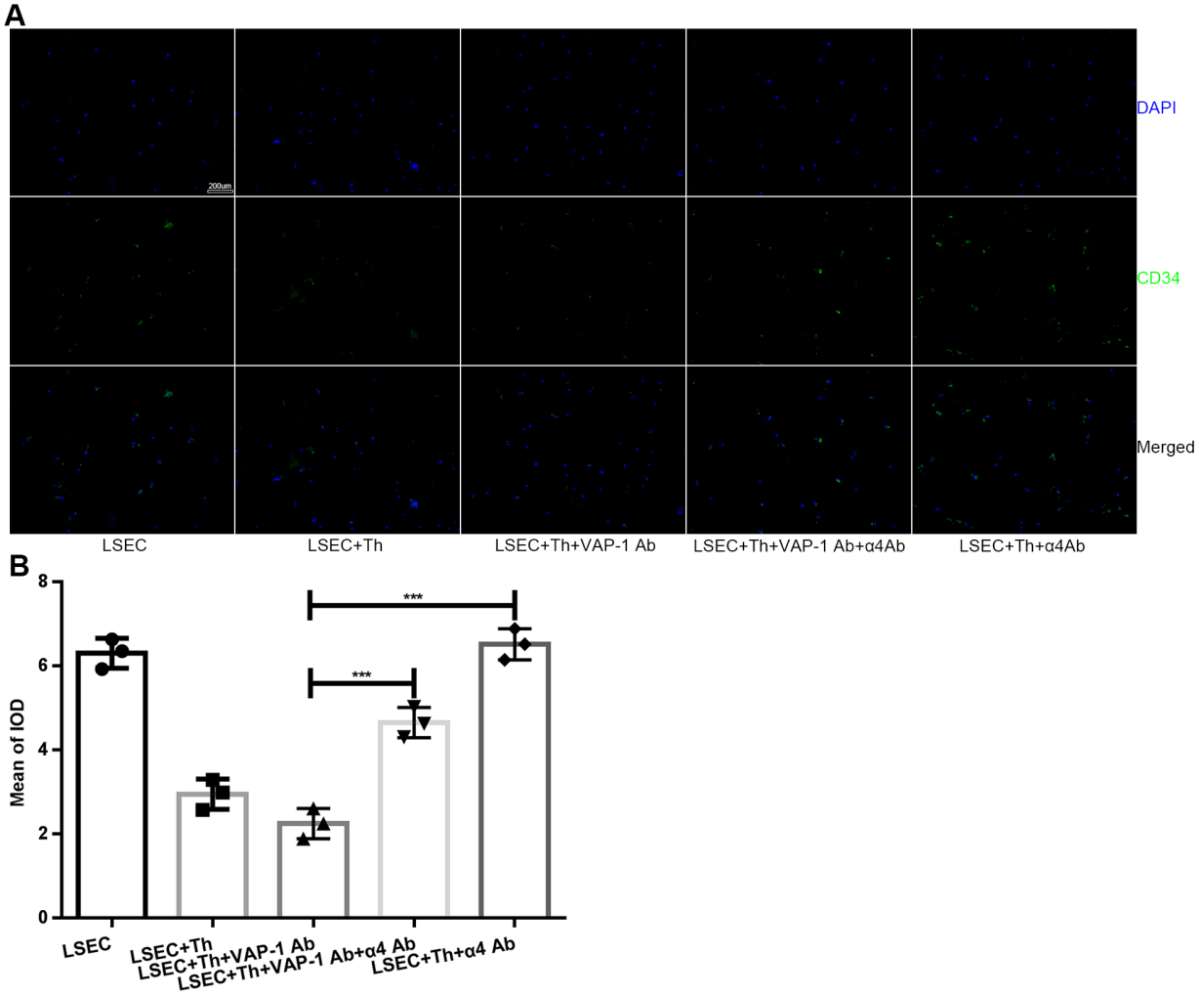
**Expression of CD34 on LSECs in each group after *in vitro* coculture for 3 h.** (**A**) Immunofluorescence staining for CD34 expression on LSECs (original magnification 100×). Anti-CD34 antibodies were labeled with Alexa Fluor 488 (green), and cell nuclei were labeled with DAPI (blue). (**B**) Quantification of CD34 expression of each group in (**A**). ***p < 0.001.

Next we cocultured isolated Th cells and LSECs from fibrotic rats in a culture plate, and collected the supernatant from each well at the 1 h, 2 h-, 3 h-, 4h- and 5 h time points. As shown in Figure 4A, the results showed that cytokine secretion by Th1 or Th2 cells began to show significant changes at approximately 2 h or 3 h after the initiation of coculture in the anti-VAP-1 group and the anti-integrin α4 group, indicating that inhibitory antibodies exerted substantial effects beginning at about the 3^rd^ hour after coculturing. Thereafter, we further investigated the ultrastructural morphological changes of LSECs in each group at the 3^rd^ hour after coculturing. SEM results showed that the addition of the anti-VAP-1 antibody can promote the reversion of LSECs from defenestration; more and larger fenestrae were found at the 3^rd^ hour after the initiation of coculture. And this structural change reflects, to some extent, the reduction of hepatic capillarization (Figure 4B, 4C). However, inhibition by the addition of the anti-integrin α4 antibody delayed the reversal of defenestration (Figure 4B, 4C). Similarly, we conducted a cellular immunofluorescence assay to evaluate the expression of CD34 on isolated LSECs in each group and found that anti-VAP-1 antibody treatment markedly reduced CD34 expression relative to that in the other experimental groups (Figure 5).

### Th cells influence LSECs most likely through cytoskeletal alterations mediated by the Rho-ROCK-myosin pathway

**Figure 6:**
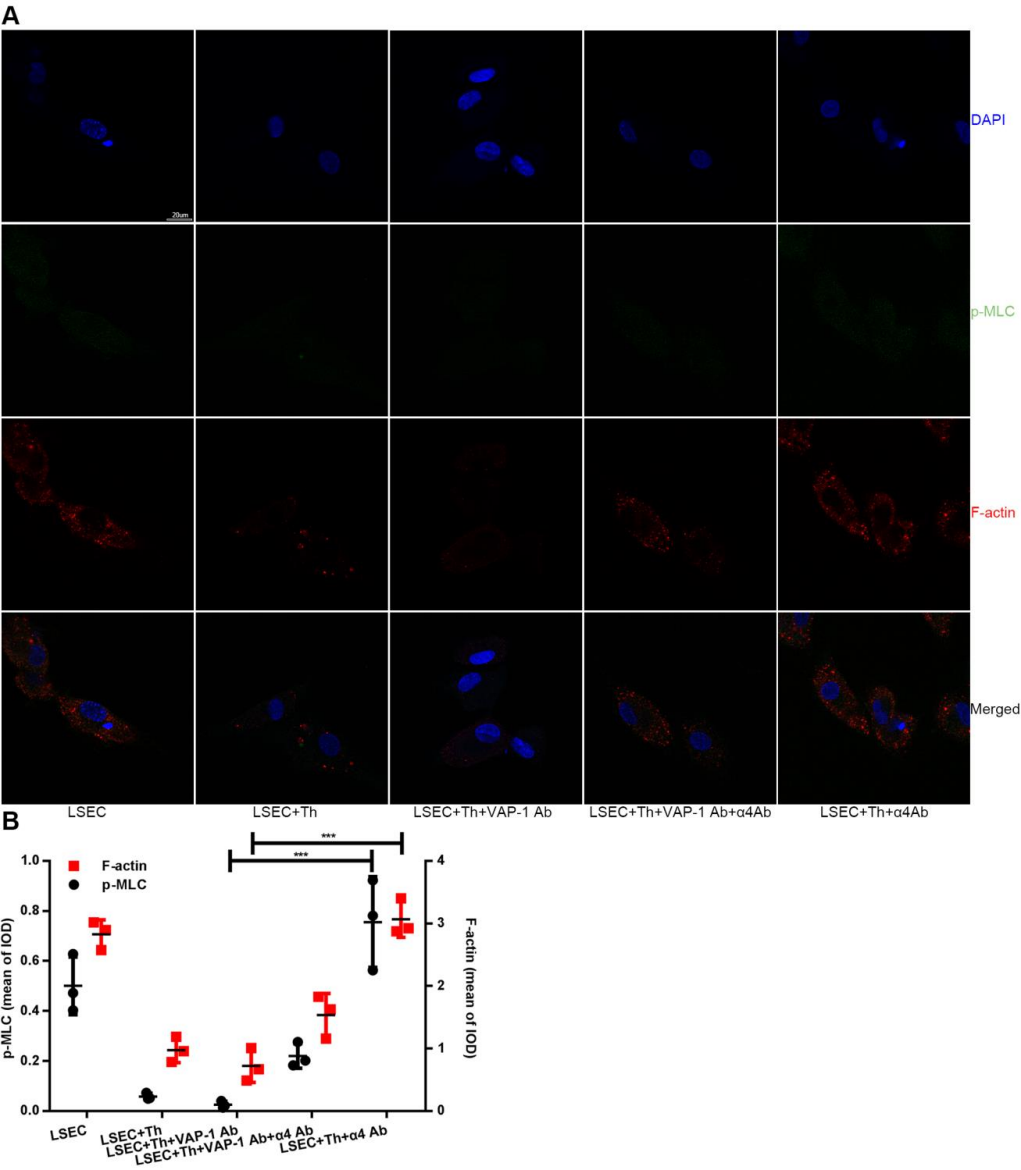
**Changes of F-actin and p-MLC in each group in *in vitro* experimental groups.** (**A**) Anti-VAP-1 antibody treatment induces a loss of stress fibers and p-MLC in isolated LSECs after coculture for 3 h, however anti-integrin α4 antibody treatment produces the opposite effect. F-actin (red) staining with Alexa Fluor 555 and p-MLC (green) staining with Alexa Fluor 488 were examined by confocal immunofluorescence microscopy. Nuclei (blue) were counterstained with DAPI. The scale bar denotes 20 μm. (**B**) Quantification of F-actin and p-MLC expression of each group in (**A**). ***p < 0.001.

**Figure 7:**
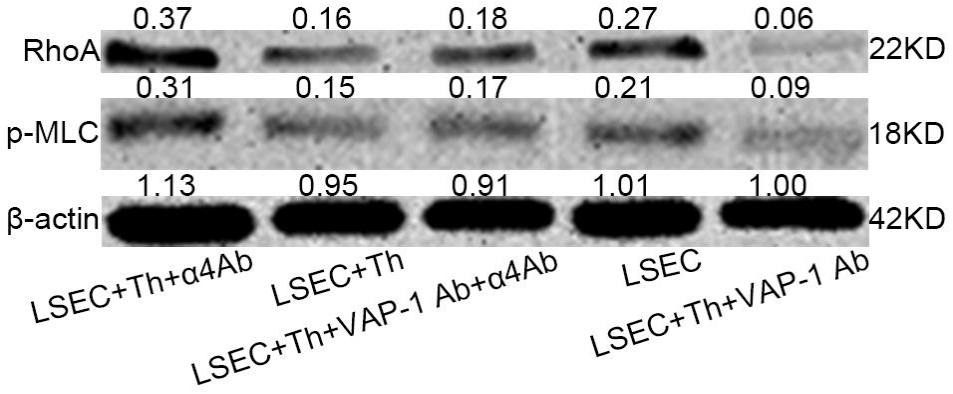
**Western blot analysis of RhoA and p-MLC levels of isolated LSECs in each group in the co-cultivation systems.** The level was higher in Lane 1 (anti-integrin α4 antibody group) than in lane 4 (pure isolated LSECs from fibrotic rats). And the level was lowest in lane 5 (anti-VAP-1 antibody group).

Previous studies have shown that SEF size was regulated by actomyosin [22, 25, 26]; besides, Rho signaling pathway was critical in modulating cytoskeleton [20–22]. Combining the results in this study that blocking interactions between Th lymphocytes and LSECs led to changes of SEF size (Figure 4B, 4C), we hypothesized that Th1/2 cells likely influenced hepatic capillarization through cytoskeletal alterations mediated by the canonical Rho-ROCK-myosin signaling pathway. To prove this reasonable hypothesis, we performed an immunofluorescence confocal microscopy assay by staining F-actin stress fibers and phosphorylated myosin light chain (p-MLC) to elucidate the mechanism underlying the morphological change of fenestrae in *in vitro* experiments. Isolated LSECs treated with the anti-VAP-1 antibody had lower p-MLC content than that in other experimental groups. In addition, a loss of stress fibers was observed in anti-VAP-1 group compared with that in other experimental groups. However, in the anti-integrin α4 group, p-MLC and F-actin stress fiber content showed the highest levels compared with other groups (Figure 6). To further confirm the light and electron microscopy results and to investigate the role of the Rho-ROCK-myosin pathway in the alteration of SEF, we performed western blot for Rho and p-MLC, the two key proteins of this signaling pathway, and found that expressions of Rho and p-MLC were lower in anti-VAP-1 antibody LSECs but relatively higher in anti-integrin α4 antibody LSECs than other groups (Figure 7).

## DISCUSSION

Previous studies have reported that CD4^+^ T cells participate in immune regulation in the liver [27, 28]. In this study, we found the importance of the balance between Th1 and Th2 cells in maintaining the process of hepatic capillarization, consistent with previous studies [11, 14, 29]. We provided evidence that inhibiting the interaction of Th1/2 cells with LSECs can affect hepatic capillarization or even liver fibrosis in different directions. Specifically, after the interaction of Th2 cells with fibrotic LSECs was inhibited, the structure of SEF could be altered, thus leading to a reduction of defenestration and even recovery of hepatic capillarization; inhibiting Th1 cells with LSECs with specific inhibitory anti-integrin α4 antibody delayed these effects. Moreover, we showed that the structural alteration of SEF was mediated by the Rho-ROCK-myosin pathway. These findings were consistent with previous observations that splenic Th2 cells promoted liver fibrosis and that actin dilatation caused the restoration of fenestrae [7, 22, 26].

**Figure 8:**
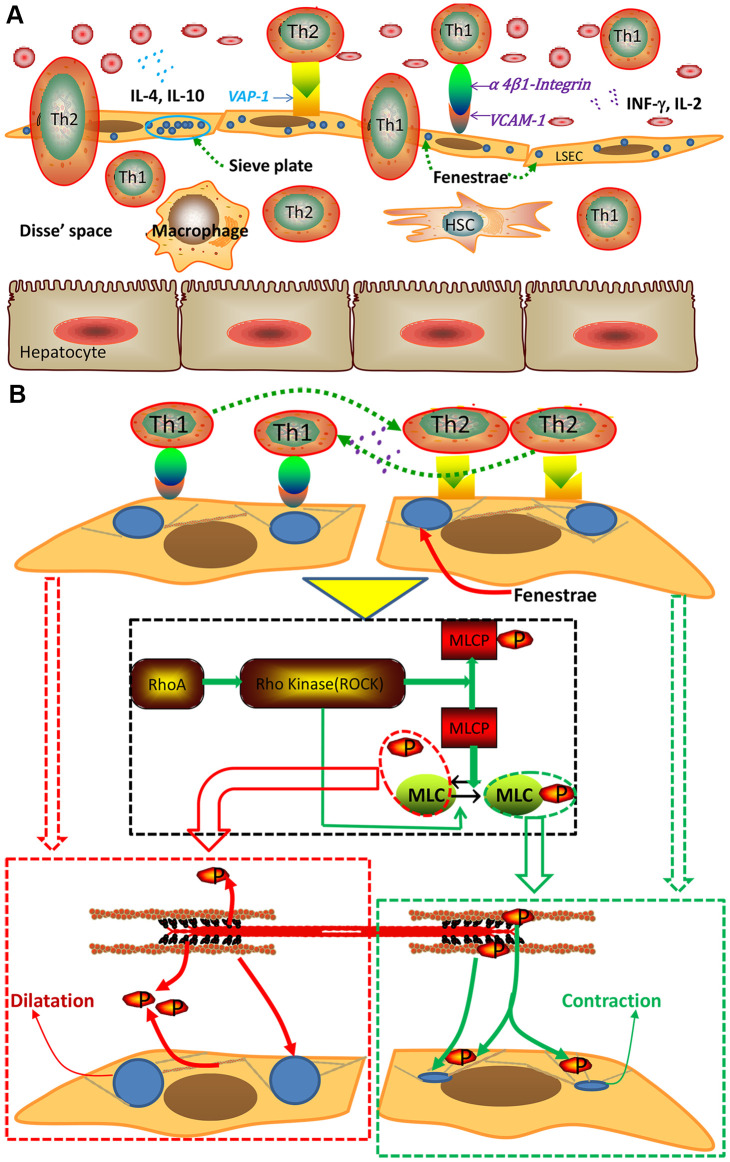
**Schematic of the interaction between Th1/Th2 cells and LSECs and the mechanism underlying this interaction.** (**A**) Interaction between Th1/Th2 cells and LSECs. (**B**) Pattern of cytoskeletal alteration mediated by the Rho-ROCK-myosin pathway. Interactions between Th1/2 cells and LSECs exerted different effects. Specifically, interactions between Th2 cells and LSECs activated the signal pathway to promote phosphorylation of myosin light chain (MLC), hence causing the contraction of cytoskeletal actin around LSECs and subsequent defenestration, however interactions between Th1 cells and LSECs exerted an opposite effect. MLCP, myosin light chain phosphatase.

The mechanism that mediated the recruitment of Th1 and Th2 cells was largely unclear for many years. The classic paradigm for leukocyte recruitment was based on a selectin-dependent mechanism until Bonder C et al*.* verified in 2005 that Th1 and Th2 cells interact with LSECs via integrin α4 and VAP-1, respectively [12]. After that, roles of integrin α4 and VAP-1 in lymphocyte recruitment in liver sinusoids were gradually established (Figure 8A). As shown in our study, blocking interactions between Th1/2 cells and LSECs with inhibitory antibodies induced different effects, which were consistent with previous studies [12, 30]. Additionally, under physiological conditions, Th1 and Th2 cells have been proven to be in dynamic balance, maintaining immune system activity. However, after the liver is affected by some chronic liver diseases, the balance is disrupted and predominantly shifts towards one direction, a process called "polarization" [7, 31]. As previously shown [7, 32], Th2 cells are crucial in fibrotic disorders by shifting the cytokine balance towards Th2 dominance; this finding was consistent with our results that inhibiting Th2 cell recruitment using the anti-VAP-1 antibody alleviated the progression of hepatic capillarization or even liver fibrosis by altering the Th2 dominance.

Fenestration, which is characterized by scattered fenestrae on the surface of LSECs, constitutes the unique phenotype of LSECs. Commonly, under long-term exposure to hepatotoxic substances, differentiated LSECs will lose their normal phenotype and transition into another state preceding fibrosis, a process called "capillarization" [33]. Then, lymphocytes recruited into sinusoids, where they interacted with different cells such as LSECs, hepatic stellate cells (HSCs) and hepatocytes to regulate progression from capillarization to liver fibrosis [34–37]. Our study revealed a similar defenestration phenomenon in the fibrotic rat model. However, the stimulus initiating these changes—in other words, the trigger of defenestration—is not yet fully known, although numerous factors, such as aflatoxin [38–40], the c-terminal fragment of thrombospondin-1 (P4N1) [41] and iron overload [42] have been reported as possible causes. Because cytoskeletal changes can alter the size of fenestrae and because the cytoskeleton is regulated by actomyosin (Figure 8B) [22, 25, 26], we explored changes in cytoskeletal signaling after preventing the interaction of Th2 cells with LSECs and found decreased Rho activation and MLC phosphorylation, which caused the subsequent dilatation of cytoskeletal actin around LSECs and alleviated hepatic capillarization (Figure 8B).

Our research still has some limitations. Firstly, changes of the Rho-ROCK-myosin pathway may not be the direct results caused by Th1/2 lymphocytes, the specific mechanism and possible intermediate medium still need to be further explored. Secondly, additional studies are still needed in the future to reveal the relevance between the Rho-ROCK-myosin pathway and another classical nitric oxide (NO)-mediated pathway [18] and to develop new drugs.

This research creatively studied the effect of blocking the recruitment and interaction of Th1/2 lymphocytes to LSECs in the fibrotic liver and explored the possible mechanism at the cytoskeletal level. We elucidated the correlation between circulating Th1/2 cells and liver sinusoids, and provided new insights into the alleviation or even regression of liver fibrosis.

## CONCLUSIONS

Taken together, our data provided evidence that Th1/2 lymphocytes interacted with LSECs through different adhesion molecules and that inhibiting these interactions influenced the progression of hepatic capillarization or even liver fibrosis to some extent, which was likely mediated by the Rho-ROCK-myosin pathway.

## MATERIALS AND METHODS

### Animals

One-month-old male wistar rats weighing 80-100 grams were used to establish the model of liver fibrosis according to a previous method [43]. Briefly, repeated intraperitoneal injection of 50% carbon tetrachloride (CCl_4_) dissolved in corn oil (0.3 ml/100 g, twice a week for 6 weeks) were administered. The animals were housed two per cage and were fed standard laboratory chow. The study was approved by the Sun Yat-sen University Animal Ethics Committee. The rats either received access to rat chow and water ad libitum until the time of surgery or received only water for 24 h prior to surgery. Anesthesia was induced by the intraperitoneal injection of 3% sodium pentobarbital solution (30 mg/kg).

### Administration of inhibitory antibodies to model rats

After the model was established, the rats were treated with corresponding inhibitory antibodies. The binding specificities of anti-VAP-1 monoclonal antibody (mAb) (174–5, mouse IgG1, Abcam, MA, USA) and anti-integrin α4 mAb (TA-2, mouse IgG1, ThermoFisher, USA) were fully demonstrated in published studies [44, 45]. A dose of 2 mg/kg anti-VAP-1 antibody was conducted for each rat every second day after the initial injection (anti-VAP-1 group); some fibrotic rats received daily injections of anti-integrin α4 antibody (5 mg/kg) (anti-integrin α4 group) or both two (co-injection group); irrelevant isotype-matched control antibody (NS1) 2mg/kg every other day was administrated to some rats as the control group (NS1). We divided the fibrotic rats into 4 experimental groups, named the anti-VAP-1 group, the anti-integrin α4 group, the combined injection (coinjection) group with anti-VAP-1 and anti-integrin α4 and the control group (NS1), and normal rats from the same batch were selected as the normal control (NC) group. Each group had at least 21 rats, and antibody diluted with sterilized physiological saline to a volume of 500 μl was injected *i.v.* via a tail vein. Then, we detected the changes of cytokine secretion, transaminase level, CD34 expression, fenestrae number, and cytoskeletal morphology in each group.

### Isolation and purification of rat LSECs

According to a previous method [46], collagenase perfusion and percoll layers of different density gradients (25% and 50%) (GE Healthcare, MA, USA) were used to isolate LSECs. After harvested, they were seeded on 12 mm coverslips (Electron Microscopy Sciences, PA, USA) coated with type I collagen (Thermo Fisher Scientific, MA, USA) at a density of 2.5×10^5^ cells/cm^2^. The purity of the cultured LSECs was > 90%, as determined by immunofluorescence assays with anti-von Willebrand factor (vWF) (11778-1-AP, Proteintech) and anti-endothelial cell antigen-1 (RECA-1) antibodies (ab9774, Abcam, UK) (Supplementary Figure 2).

### Purification of CD4^+^ T cells and differentiation of Th1 and Th2 cells

We used superparamagnetic polystyrene beads (Miltenyi Biotec GmbH, Germany) coated with a mouse anti-rat CD4 mAb (isotype: mouse IgG2a, κ; clone: OX-38) to harvest CD4^+^ T cells from spleen-derived lymphocytes, which were extracted using rat tissue lymphocyte isolation kit according to the protocol. The purity of CD4^+^ T cells was > 90%, as determined by a flow cytometry assay (Supplementary Figure 3). Then CD4^+^ T cells were stimulated to differentiate Th1 or Th2 with stimulating factors for 6 days, followed by published studies previously [12, 47–49]. Briefly, for Th1 polarization, 10 ng/ml recombinant interleukin-2 (rIL-2), 5 ng/ml rIL-12 and 30 μg/ml anti-IL-4 were used; for Th2 polarization, 10 ng/ml rIL-2, 40 ng/ml rIL-4, 30 μg/ml anti-IL-12 and 30 μg/ml anti-IFN-γ were used (all cytokines and antibodies against cytokines were purchased from PeproTech Inc., NJ, USA). After the differentiation into Th1 and Th2 cells, we detected the levels of IFN-γ and IL-4, IL-5 and IL-13, which could be viewed as the signature cytokines of Th1 and Th2 respectively [50] to identify the purity of Th1 and Th2 cells (Supplementary Figure 4).

### Coculture of LSECs with Th cells

Isolated LSECs from fibrotic rats were seeded at a density of 1×10^6^ cells/ml in culture plates, and Th1 and Th2 lymphocytes (at an adjusted concentration of 9×10^4^ cells/ml per type) were then added into the culture system. After adding antibodies, we assessed cytokine level, fenestrae structure and protein level of the Rho-ROCK-myosin pathway in each group. Each assay was conducted at least 3 times, and 20 μg of each inhibitory antibody was added to each well.

### Enzyme-linked immunosorbent assay (ELISA)

The concentrations of IFN-γ, IL-4, IL-5, IL-13, ALT and AST were measured by ELISA (R&D Systems, USA) [51].

### Hematoxylin-eosin (HE)/masson staining

HE and Masson staining (all reagents were purchased from Beyotime Institute of Biotechnology, Shanghai, China) were conducted as a published study [52]. The liver fibrosis score was evaluated according to the method described in a previous study [53].

### Immunohistochemical staining

Paraffin-embedded and formalin-fixed samples were cut into 4-μm-thick sections, which were then processed for immunohistochemical staining [54].

### Hydroxyproline content measurement

Liver tissues were homogenized in ice-cold distilled water (1 ml). The subsequent steps were conducted according to a previous protocol [7]. The results are shown as nanograms of hydroxyproline per milligram of liver tissue.

### Scanning electron microscopy (SEM)

The structure of SEF was observed by SEM [55]. Briefly, samples were fixed with 2.5% glutaraldehyde and postfixed with 1% osmium tetroxide. Then, samples were critical point dried, sputter coated with gold, and finally examined with a JSM-T200 SEM (JEOL, Tokyo, Japan).

### Image analysis

Measurements of the fenestrae number and porosity (total fenestration area) were performed using Image J software [56]. The index fenestrae/μm^2^ was calculated according to the total fenestrae number normalized to the cell area per image. To calculate the porosity/μm^2^, the fenestrated area in each image was summed to yield the total area, which was then normalized to the total area of the cell. For each experiment, at least five images were analyzed per sample.

### Transmission electron microscopy (TEM)

We used TEM to examine the changes of SEF and the basement membrane [25].

### Confocal immunofluorescence microscopy

Specimens were fixed, stained with an anti-phosphorylated myosin light chain (p-MLC) rabbit mAb (#3671, CST, MA, USA) and an anti-F-actin mouse mAb (ab205, Abcam), and then incubated with the corresponding secondary antibodies (Alexa Fluor 488 and Alexa Fluor Plus 555 (ThermoFisher), respectively) at room temperature for 1 h. Samples were visualized using a laser scanning confocal microscope (LSM780, Zeiss).

### Western blot analysis

Total protein of cultured LSECs was separated on 12% SDS-PAGE gels and transferred to nitrocellulose membranes (0.45 μm or 0.2 μm). The levels of RhoA and p-MLC were detected with an enhanced chemiluminescence (ECL) kit (#6883S, CST, MA, USA) according to the instructions. The antibodies used were as follows: an anti-RhoA primary antibody (ab54835, Abcam, MA, USA) and an anti-p-MLC primary antibody (#3671, CST, MA, USA). An anti-β-actin antibody (Abcam, MA, USA) was used as a control.

### Statistical analysis

The data are shown as the means ± standard deviation (SD). Differences between groups were analyzed using Student’s *t*-test if only two groups were compared or using one-way analysis of variance (ANOVA) if more than two groups were compared. All statistical tests were two-tailed. All experiments were performed at least three independent times. *P* < 0.05 was considered statistically significant.

## Supplementary Material

Supplementary Figures
