# Supplementary material for: Inhibiting Th1/2 cells influences hepatic capillarization by adjusting sinusoidal endothelial fenestrae through Rho-ROCK-myosin pathway
Source: Aging (Albany NY). 2021 Feb 1;13(4):5069–86. doi: 10.18632/aging.202425 (PMC7950229; doi:10.18632/aging.202425)
Supplement: Supplementary Figures [file aging-13-202425-s001.pdf]

SUPPLEMENTARY FIGURES

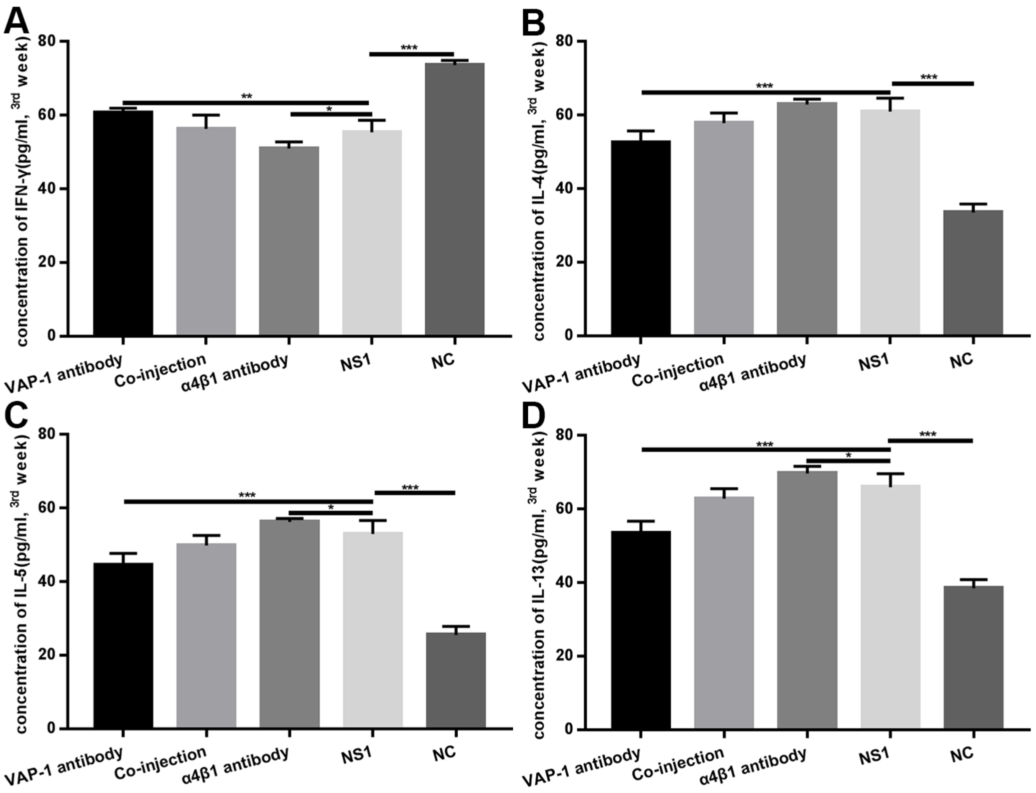

Supplementary Figure 1. Comparison of cytokines in each experimental group to the NS1 group at the 3rd week after injection. Analysis of cytokine IFN-γ (**A**), IL-4 (**B**), IL-5 (**C**) and IL-13 (**D**) in each group. *p< 0.05, **p< 0.01, ***p< 0.001.

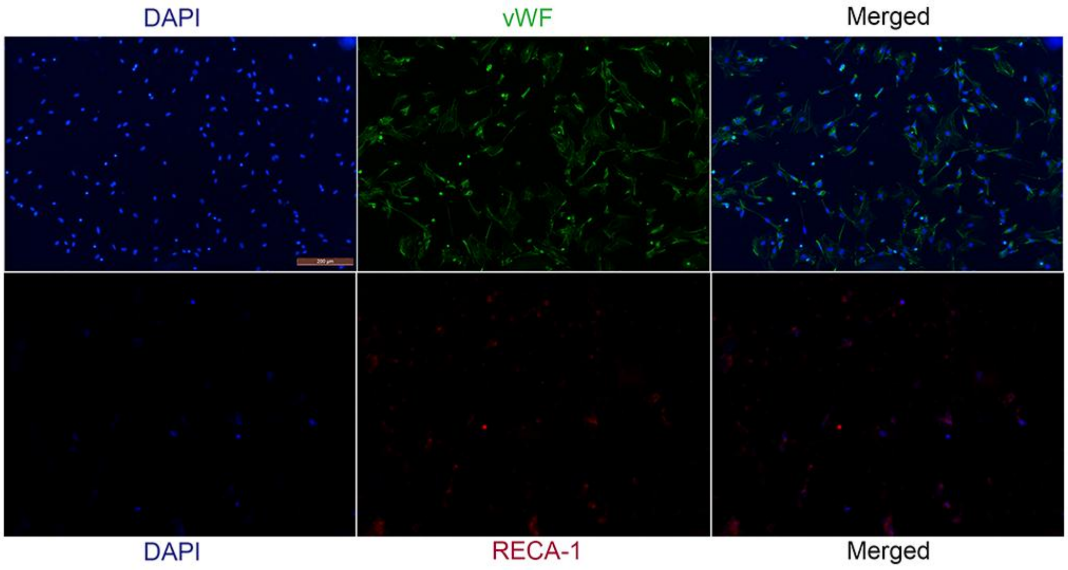

Supplementary Figure 2. Purity identification of isolated LSECs. Immunofluorescent staining for von Willebrand factor (vWF) and endothelial cell antigen-1 (RECA-1) in LSECs (original magnification 100×). vWF was labeled with Alexa Fluor 488 (green) and RECA-1 was labeled with Alexa Fluor 555 (red) and cell nucleus was labeled DAPI (blue).

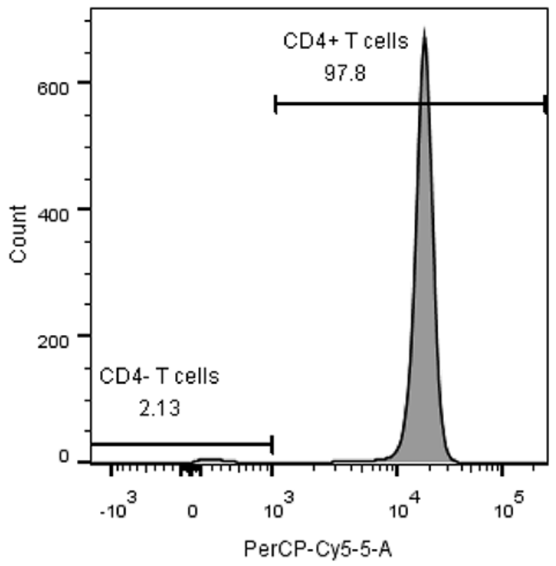

Supplementary Figure 3. Purity of isolated CD4$^+$ T cells by flow cytometry was 97.8%.

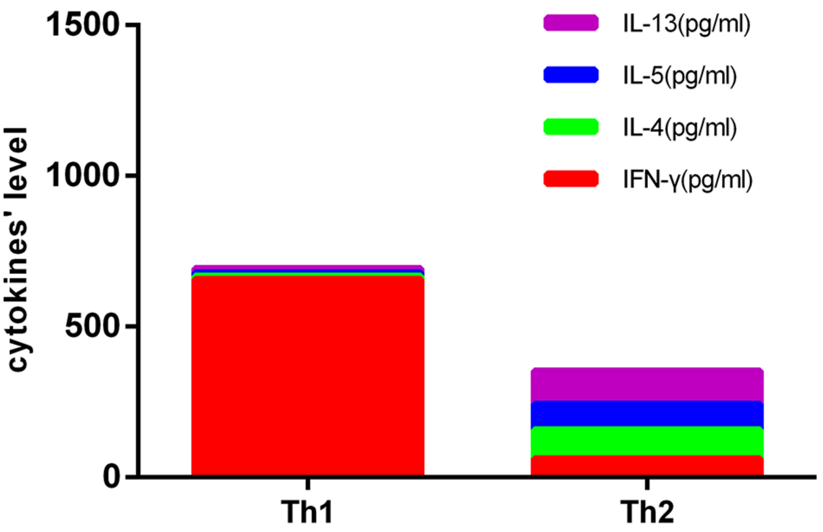

Supplementary Figure 4. After differentiation, Th1 and Th2 began to produce a large number of their own signature cytokines, despite little cross secretion.
